# 16. A rare case of CNS demyelination with anti-TNF treatment

**DOI:** 10.1093/rap/rky033.008

**Published:** 2018-09-20

**Authors:** Mithun Chakravorty, Shaza Obaid

**Affiliations:** Rheumatology, East Midlands, Lincoln, UNITED KINGDOM

**Introduction:** Anti-tumour necrosis factor (TNF) alpha treatment is commonly used in rheumatology, gastroenterology and other specialties to control conditions such as ankylosing spondylitis (AS), psoriatic arthritis (PsA), rheumatoid arthritis (RA) and Crohn’s disease. Examples include etanercept, adalimumab, infliximab, golimumab and certolizumab. Their effectiveness is often superior to conventionally used disease-modifying anti-rheumatic drugs (DMARDs) and the side-effect profile thought to be relatively safe. However, clinical trials using anti-TNF to treat multiple sclerosis worryingly demonstrated increased disease activity rather than clinical improvement. Although etanercept was not used in these trials, there have been rare reports of it causing both central nervous system (CNS) and peripheral nervous system demyelination. This case once again highlights this concerning complication of anti-TNF therapy.

**Case description:** A 58 year old woman presented to the rapid access eye clinic with sudden onset diplopia whilst driving. She did not experience any headaches, vomiting or additional focal neurological symptoms. Her past medical history included a delayed diagnosis of AS in 2016 on a background of longstanding back pain, which was well controlled with subcutaneous etanercept 50 mg weekly. She had been treated with etanercept for over a year, with one short break in treatment due to transient leucopenia. Clinical examination revealed a left 6th cranial nerve palsy with visual acuity 6/6 bilaterally, normal pupillary response, fundus examination and intraocular pressures. There was no other neurological deficit in cranial nerve or peripheral neurological examinations. Urgent CT head was unremarkable but her symptoms persisted. Her etanercept was stopped in the rheumatology clinic with urgent MRI brain and neurology review arranged. The MRI scan demonstrated an area of high signal intensity in the dorsal left pons, with additional small foci adjacent to the posterior horn of the left lateral ventricle; consistent with demyelination. She was treated by neurology for a clinically isolated syndrome with oral methylprednisolone 500 mg for five days. Her diplopia completely resolved after two weeks. She is currently awaiting to have a lumbar puncture and MRI brain scan with gadolinium. Alternative treatments are being considered for her AS.

**Discussion:** There was high clinical suspicion of demyelination given the use of anti-TNF treatment and new, persistent neurological symptoms. The CT head was done prior to clinic review and did not show an alternative cause e.g. space-occupying lesion. It was therefore felt necessary to suspend treatment until the more sensitive MRI scan was performed with neurology input. Once the likelihood of demyelination was confirmed, anti-TNF was stopped indefinitely and further treatment would be reviewed later given the AS was clinically quiescent. Initial animal studies demonstrated beneficial effects of anti-TNF therapy in multiple sclerosis (MS) and subsequent human trials used infliximab and lenercept in patients with MS. However, the results were staggering as both treatments caused worsening of disease. Moreover, there have been an increasing number of demyelinating events with the growing use of anti-TNF. Kemanetzoglou et al. found 122 published cases of CNS demyelinating events in patients treated with anti-TNF between January 1990 and August 2016. The mean time from exposure of anti-TNF to onset of neurological symptoms was 17.6 months. The mean age of onset was 45 years; later than typical presentations of MS but similarly there was a female preponderance - 61% of all reported cases. Interestingly, only three patients had a reported family history of MS. 50% of patients had RA with only 11% having AS. 16% had PsA, 8% had Crohn’s disease and the remainder had other inflammatory conditions. The majority (47%) were treated with etanercept, followed by infliximab in 41% of cases. 16% were treated with adalimumab and 1% had golimumab. Three patients received more than one anti-TNF drug. Recovery after initial therapy was complete in 36% of patients but only partial in 21%. There was no resolution in symptoms in 28% of patients and three patients died - two with PML and one with MDE.

**Key Learning Points:** Although rare, there should be a high index of suspicion of demyelination in all patients treated with anti-TNF therapy (including biosimilars) who develop persistent neurological symptoms. Isolated 6th cranial nerve palsy is often a false localising sign but in this case was caused by demyelination in the pons; where the cranial nerve nucleus was located. Family history of MS does not appear to be associated with significant increased risk of demyelination with anti-TNF treatment. Just over a third of patients obtain full recovery after initial therapy. Data for longer follow up periods appears to be lacking.

**Disclosure: M. Chakravorty:** None. **S. Obaid:** None.

